
JOURNAL INFORMATION
==============================
NLM Title Abbreviation: ScientificWorldJournal
Journal ID: ScientificWorldJournal
NLM Journal ID: 101131163
Journal ID: TSWJ

The Scientific World Journal

ISSN: 2356-6140
EISSN: 1537-744X
Publisher: Wiley

ARTICLE INFORMATION
==============================
PMCID: PMC6009313
PMID: 29973832
DOI: 10.1100/tsw.2002.57
Article ID: 237814
Article version: 1
Subjects: Short Report

Eukaryotic Targets of a Bacterial Protein Kinase Virulence Factor

Schesser Kurt 1 2 *
Dukuzumuremyi Jean-Marie 2
Bennett Matt 2
Bartra Sara 2
Rosqvist Roland 3
Wolf-Watz Hans 3
Department of Microbiology & Immunology, University of Miami School of Medicine, P.O. Box 016960 (R-138), Miami, FL 33101, USA
Department of Cell & Molecular Biology, Lund University, Lund, Sweden
Department of Cell & Molecular Biology, Umeå University, Umeå, Sweden
*Kurt Schesser: kschesser@med.miami.edu
Publication date: 2002
Electronic publication date: 2002 Jul 9
Volume: 2
First page: 121
Last page: 122
Copyright: Copyright © 2002 Kurt Schesser et al.
Copyright year: 2002
License: This is an open access article distributed under the Creative Commons Attribution License, which permits unrestricted use, distribution, and reproduction in any medium, provided the original work is properly cited.
License URL: https://creativecommons.org/licenses/by/3.0/

Funding: Swedish Medical Research Council Swedish Foundation of Strategic Research Department of Microbiology and Immunology University of Miami

==============================
Advances in Gene Technology: The Genome and Beyond –
Structural Biology for Medicine (Proceedings of the 2002 Miami
*Nature Biotechnology* Winter Symposium)
*TheScientificWorld* 2002, 2(S2), 121–122
ISSN 1532-2246; DOI 10.1100/tsw.2002.57

### EUKARYOTIC TARGETS OF A BACTERIAL PROTEIN KINASE VIRULENCE FACTOR

Kurt Schesser[1,2,*], Jean-Marie Dukuzumuremyi[2], Matt Bennett[2], Sara Bartra[2], Roland Rosqvist[3], and Hans Wolf-Watz[3]
[1]Department of Microbiology & Immunology, University of Miami School of Medicine, P.O. Box 016960 (R-138), Miami, FL 33101; [2]Department of Cell & Molecular Biology, Lund University, Lund, Sweden; [3]Department of Cell & Molecular Biology, Umeå University, Umeå, Sweden
*kschesser@med.miami.edu

**INTRODUCTION.** Several animal- and plant-interacting Gram-negative bacteria possess the so-called type III secretion system or TTSS, which functions to inject proteins (or 'effectors') directly into eukaryotic cells[1]. TTSS effectors play important roles in a variety of host-microbe interactions including *Rhizobium*-mediated root nodulation, plant hypersensitivity reactions, and in animals, modulating immune responses. The pathogenic yersiniae (*Yersinia pestis*, *Y. pseudotuberculosis*, and *Y. enterocolitica*) inject five TTSS effectors all of which are required for full virulence in the mouse model. As might be expected since they function within host cells, *Yersinia*'s TTSS effectors possess clear eukaryotic-like protein domains or motifs. One of these effectors, the *Yersinia* protein kinase A (YpkA), contains the canonical 11 subdomains (I-XI) characteristic of eukaryotic ser/thr protein kinases with a highly conserved catalytic core sequence. In addition to the amino-terminally located kinase domain, YpkA, also contains in its carboxyl-terminal region sequences that resemble eukaryotic RhoA-binding domains.

**RESULTS AND DISCUSSION.** We have previously shown by flow cytometry that the expression of TTSS effectors increase in individual bacteria following contact with cultured macrophage-like cells[2]. In a cell culture infection assay, a strain of *Y. pseudotuberculosis* expressing an enzymatic inactive variant of YpkA ($YpkA_{D270A}$) was greatly attenuated in its ability to proliferate compared to the wild-type strain when incubated with eukaryotic cells. To identify eukaryotic proteins that physically interact with YpkA, we performed yeast two-hybrid screening using YpkA's kinase domain as bait. In a library prepared from human spleen, we discovered that the calcium- and integrin-binding (CIB) protein interacts with YpkA and have gone on to show that the YpkA-CIB association occurs in an *in vitro* protein interaction assay. We are currently testing whether CIB (a.k.a. 'calmyrin'), a peripheral membrane protein[3,4] in fact localizes YpkA to the inner face of the plasma membrane following its injection into eukaryotic cells[5]. We have also found that YpkA's kinase activity was not required in a cell morphology assay in which YpkA induces HeLa cells to undergo retraction. Instead the carboxyl-terminal region of YpkA, which contains sequence similarity to eukaryotic RhoA-binding domains, is required for YpkA-mediated morphological changes of eukaryotic cells[5]. YpkA does indeed interact with RhoA (and Rac but not Cdc42) in protein interaction assays, but interestingly, unlike eukaryotic RhoA-binding proteins, with which it shares sequence similarity, YpkA binds both the activated GTP-bound form of RhoA as well as inactive GDP-bound RhoA.

Although the significance of CIB and RhoA targeting by YpkA, both in terms of the cell biology and, in a larger sense, pathogenesis, is currently unclear, we believe that characterizing these events will eventually lead us to an understanding of how this virulence factor promotes bacterial proliferation vis-à-vis the eukaryotic host cell.

**ACKNOWLEDGMENTS.** Supported by the Swedish Medical Research Council, the Swedish Foundation of Strategic Research, and the Department of Microbiology and Immunology, University of Miami School of Medicine.

**REFERENCES**

1. Hueck,C.J. (1998) Microbiol. Mol. Biol. Rev. 62, 379–433.
2. Bartra, S., Cherepanov, P., Forsberg, Å., and Schesser, K. (2001) BMC Microbiol 1, 22.
3. Naik, U.P., Patel, P.M., and Parise, L.V. (1997) J. Biol. Chem. 272, 4651–4654.
4. Stabler, S.M., Ostrowski, L.L., Janicki, S.M., and Monteiro, M.J. (1999) J. Cel Biol. 145, 1277–1292.
5. Dukuzumuremyi, J.-M., Rosqvist, R., Hallberg, B., Åkerstrom, B., Wolf-Watz, H., and Schesser, K. (2000) J. Biol. Chem. 275, 35281–35290.
